# Stockton’s Dental Intelligencer

**Published:** 1845-12

**Authors:** 


### STOCKTON'S DENTAL INTELLIGENCER.

This journal has reached its second volume, the first number of which making 22 pages, we received a few days since in exchange. It is conclusive proof of the general interest felt in dentistry, that two periodicals devoted to that branch of the art can be supported in this country. Among the articles contained in this number we find one of Mr. Liston's lectures, and altogether it is made up of matter interesting to every practitioner. Y.

